# The Role of the JAK-STAT Signaling Pathway in the Protective Effects of Hepatic Ischemia Post-conditioning Against the Injury Induced by Ischemia/Reperfusion in the Rat Liver

**DOI:** 10.34172/apb.2024.003

**Published:** 2023-07-19

**Authors:** Neda Ghasemi Pour Afshar, Hossein Ali Arab, Akram Vatannejad, Ghorbangol Ashabi, Ali akbar Golabchifar

**Affiliations:** ^1^Department of Comparative Biosciences, Faculty of Veterinary Medicine, University of Tehran, Tehran, Iran.; ^2^Department of Physiology, Faculty of Medicine, Tehran University of Medical Sciences, Tehran, Iran.

**Keywords:** Apoptosis, Ischemic post-conditioning, Ischemia/reperfusion, JAK inhibitor, Liver

## Abstract

**Purpose:**

Hepatic ischemic post-conditioning (IPOC) is shown to protect the liver from injury induced by ischemia/reperfusion (IR). However, the mechanism underlying this protection has remained elusive. The present study aimed to investigate the role of the *interleukin 6-Janus* kinase-signal transducers and activators of transcription (IL-6-JAK-STAT) pathway in the protective effect of hepatic IPOC against the IR-induced injury in the liver.

**Methods:**

Twenty-five rats were randomly divided into 5 groups of (1) sham-operated, (2) IR, (3) IR+hepatic IPOC, (4) IR+tofacitinib (TOFA), and (5) IR+TOFA+hepatic IPOC. The changes induced by IR and the effects of different treatments were assessed by enzyme release, histopathological observations, the serum level of IL-6, and the occurrence of apoptosis detected via the expression of the Bax/Bcl-2 ratio.

**Results:**

The hepatic IPOC improved the liver injury induced by IR as shown by histological changes, reduction of IL-6 level, aspartate aminotransferase (AST), and alanine aminotransferase (ALT) compared to the IR group (*P*<0.001, *P*<0.05, *P*<0.05, respectively). There was also downregulation of the Bax/Bcl2 ratio in the rats exposed to IR+hepatic IPOC compared with those in the IR group (*P*<0.05). However, TOFA, an inhibitor of JAK-STAT activity, inhibited the protective effect of hepatic IPOC.

**Conclusion:**

It suggests that the protective effect of hepatic IPOC against IR-induced injury may be mediated by activating the IL-6-JAK-STAT pathway.

## Introduction

 Hepatic ischemia/reperfusion injury (IRI) is a condition in which the reperfusion of the liver paradoxically augments the damage induced during ischemia. This phenomenon, which occurs by cyclic interruption of short-term blood flow followed by the reperfusion of the ischemic tissues, is usually associated with microcirculation disturbances, inflammatory and oxidative stress processes.^1^ Hepatic IRI is observed after the surgery of the liver conducted in many pathological conditions such as liver transplantation, benign and malignant tumors, abscesses, and cysts.^1,2^ The IRI of the liver is associated with different changes including intracellular acidosis, ATP depletion, mitochondrial dysfunction, increased intracellular Ca^2+^, reactive oxygen species (ROS) generation, and cytokines release.^3^ The excessive production of ROS and the depletion of antioxidant capacity can lead to oxidative stress. The oxidative stress can induce a chain of alterations in the hepatocytes which is finally leading to ATP reduction, necrosis, and apoptosis.^4,5^

 Different pharmacological and surgical procedures including the ischemic post-conditioning (IPOC) have been used to attenuate the liver injury induced by ischemia/reperfusion (IR).^3^ IPOC is defined as a brief IR cycle applied to the ischemic tissues before the commencement of reperfusion.^6^ The IPOC has been shown to protect the cells against apoptosis through both inhibiting the opening of mitochondrial permeability transition pores (*MPTP*) and downregulation of the Bax/Bcl-2 ratio.^7,8^ Apoptosis as an important cell death mechanism is regulated by different signaling pathways including the JAK-STAT signaling pathway.^9^ The JAK-STAT pathway is an important signaling pathway involved in different cellular processes including migration, cell proliferation, inflammation, and apoptosis.^10^ There are four JAKs and seven STAT protein families among them, the STAT3 regulates respiration and mitochondrial-mediated apoptosis and inhibits the *MPTP* opening.^11^ The JAK-STAT pathway transmits extracellular signals from cytokine receptors to the nucleus via increased STAT3 phosphorylation. This can lead to the reduction of apoptosis by increasing Bcl-2 anti-apoptotic protein expression and decreasing Bax apoptotic protein expression.^12^ However, tofacitinib as a well-known JAK1/JAK3 inhibitor, induces apoptosis in the cells via downregulation of Bcl 2, upregulation of Bax, and cleaved caspase 3.^13^

 Studies show that IPOC is able to reduce IR injuries in a variety of tissues including the lung,^14^ kidney,^15^ heart,^16^ intestine,^17^ brain,^18^ and liver.^19^ Goodman et al have shown that the protective effects of post-conditioning against IR injuries are mediated via activation of the JAK-STAT pathways in the isolated mouse heart.^20^ However, there is not enough evidence to show the possible role of the JAK-STAT signaling pathway in the protective effects of IPOC against the hepatic injury induced by IR. This study is designed to investigate whether IPOC could alleviate apoptosis and inflammation in hepatic cells possibly through the IL-6-JAK-STAT signaling pathway in the rats exposed to hepatic IR.

## Materials and Methods

###  Animals and experimental groups 

 Male Wistar rats (weighing 250-300 g) were purchased from the institutional laboratory of the animal breeding center. They were kept under a 12-h dark/light cycle in a temperature-controlled environment (23 ± 2 °C) with a standard diet and water ad libitum for 7 days. Twenty-five rats have randomly divided into 5 groups; (1) sham-operated control, (2) IR exposed to ischemia followed by reperfusion, (3) IR + hepatic IPOC underwent the same procedure as the 2nd group except that IPOC was conducted before reperfusion, (4) IR + tofacitinib (TOFA) that received TOFA before reperfusion and (5) IR + TOFA + hepatic IPOC with the same procedure of the previous group except that the rats were exposed to IPOC. All procedures conducted on the animals were in compliance with the *guidelines* of the National Institutes of Health for the use of experimental animals.

###  The surgery and experimental protocol 

 Rats were fasted around 12 hours prior to surgery and they were anesthetized with 80 mg/kg ketamine (Rotexmeica, Germany) plus 10 mg/kg xylazine (Alfasan, Netherlands). The sham-operated group underwent all surgical procedures except that they do not expose to hepatic vascular ligation. The method used to induce hepatic IR is previously described.^21^ The lobar ischemia was induced by ligating the portal triad (*portal vein,* hepatic artery, and bile ductile) for 60 minutes and the reperfusion was followed for 24 hours. In the third group, after exposing the liver to 60 minutes ischemia, the hepatic post-conditioning was induced by 4 cycles of 30-second brief reperfusion followed by 30-second reocclusion before the commencement of reperfusion.^22^ The tofacitinib (Biology fermentation Company) diluted into DMSO was injected via peritoneal to the fourth group with a dose of 15 mg/kg (total 1 mL volume) 15 minutes before commencement of reperfusion. In the fifth group, tofacitinib as a JAK/STAT inhibitor was used to probe the role of this signaling pathway in the protection induced by hepatic post-conditioning against IR injury.

###  Blood and hepatic tissue sampling

 At the end of each experiment, the blood sample was taken from the rat’s heart after anesthetizing the animal with ketamine plus xylazine. The serum was separated by centrifugation at 3000 RPM, for 10 min at 20 °C and stored at -80 °C for further analysis. After the blood sampling, the animal was immediately sacrificed and the tissue samples were collected from the median and left lobes of the liver. The tissue samples were divided into 2 parts; one part was homogenized immediately and was kept at -80 °C for measurement of anti-apoptotic protein Bcl-2 and apoptotic protein Bax. The second part of the tissues was fixed with a 10% buffered formalin solution, embedded in paraffin, and then stained with hematoxylin-eosin (H&E). A light microscope was used to observe the histopathological changes induced in the hepatic tissues by different treatments.^23^

###  Biochemical analyzing

 The serum level of IL-6 was measured using DuoSet ELISA kits, according to the manufacturer’s instruction (R&D Systems). The serum levels of alanine aminotransferase (ALT) and aspartate aminotransferase (AST), as indicators of hepatic damage, were measured using a clinical automated chemistry analyzer (Hitachi). Serum data obtained from the analysis of enzymes release was reported as international units/liter (IU/L).

###  Western blotting

 Homogenized hepatic tissue (0.3 g) was lysed using radio-immunoprecipitation assay lysis (RIPA) buffer supplemented with a protease inhibitor cocktail. Protein concentrations were determined according to the Bradford protein assay.^24^ Equal amounts of proteins samples were separated by 12% SDS-PAGE gel and fractionated proteins were transferred onto the polyvinylidene fluoride (PVDF) membrane (Amersham Biosciences, Piscataway, NJ, USA). The membranes were blocked with bovine serum albumin (BSA) and then probed with Bax and Bcl-2 primary antibodies [diluted 1/1000, Cell signaling Technology Co (New York, USA)] overnight at 4 °C. The mixture was probed with a secondary antibody [diluted 1/3000, Cell Signaling Technology Co (New York, USA)] for 60 minutes at room temperature. Immunoreactive polypeptides were detected by chemiluminescence using enhanced ECL reagents and followed by exposure to radiographic film. We used beta-actin [diluted 1/1000, Cell Signaling Technology (Co. New York, USA)] as an internal protein control. The Western blotting was repeated three times. The density of protein bands was quantified via ImageJ analysis software (National Institutes of Health, Bethesda, MD, USA).

###  Statistical analysis

 Statistical analyses were performed using GraphPad Prism Software Version 9.0.0 (121). All data are expressed as the means ± SD. The comparisons of different experimental groups were performed using a one-way analysis of variance (ANOVA) followed by Tukey’s multiple comparisons test. Statistical significance difference was accepted at *P* < 0.05.

## Results and Discussion

###  Changes in the enzymes release and the level of IL-6 

 The present study was designed to investigate the possible role of the IL-6-JAK-STAT signaling pathway in the protective effect of hepatic IPOC against IR-induced injury in the rat liver. We used enzyme release as complementary markers to confirm the protective effects of IPOC against the hepatic IRI. The changes in the liver function were assessed by analyzing the serum levels of AST and ALT after 24 hours of reperfusion. The data obtained from the analysis of enzymes release in different experimental groups are compared in Table 1. As the table shows, the serum levels of AST and ALT in the group exposed to IR were significantly increased from 34.08 ± 13.81 and 13.94 ± 1.88 to 699.1 ± 242.5 and 552.7 ± 267.1 respectively, compared to the control group (P˂0.0001). However, the release of AST and ALT significantly decreased in the IR + hepatic IPOC, IR + TOFA, and IR + TOFA + hepatic IPOC groups compared to the IR group (*P*˂0.01, Table 1).

**Table 1:** Changes in the serum levels of ALT and AST in different experimental groups

**Groups**	**Number**	**ALT (IU/L)**	**AST (IU/L)**
Control	5	13.94 ± 1.88	34.08 ± 13.81
IR	5	552.7 ± 267.1*	699.1 ± 242.5*
IR + IPOC	5	90.72 ± 11.85#	118.1 ± 23.28#
IR + TOFA	5	247.2 ± 27.32*#	309.90 ± 22.7*#
IR + TOFA + IPOC	5	180.22 ± 19.97#	211.4 ± 31.24#

Note: Data are presented as Mean ± SD that are obtained from at least 5 experiments in each group. IR, ischemia/reperfusion; IPOC, Ischemic post-conditioning; TOFA, Tofacitinib. *P < 0.0001 vs Control group; ^#^*P* < 0.01 vs IR group.

**Figure 1:**
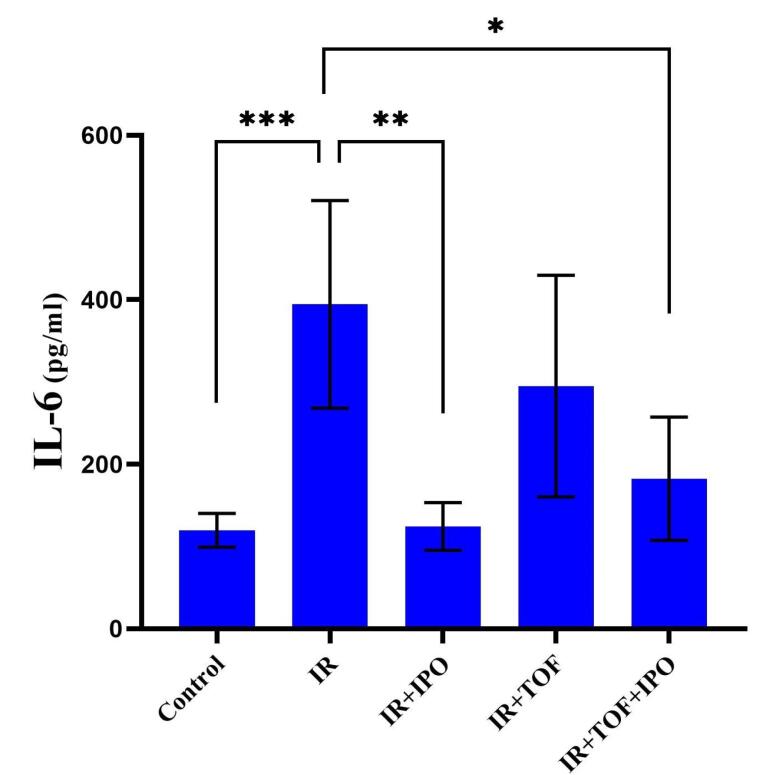
Comparison of IL-6 level between different experimental groups exposed to different treatments of sham-operation (control), ischemia/reperfusion (IR), IR plus ischemic post-conditioning (IPOC), IR plus an inhibitor of JAK/STAT, tofacitinib (TOFA), and IR plus TOFA plus IPOC. The data are expressed as the mean ± SD. * *P* < 0.05, ** *P* < 0.01, *** *P* < 0.001. IR, ischemia/reperfusion; IPOC, Ischemic post-conditioning; TOFA, Tofacitinib

 As represented in Figure 1, the level of IL-6 was also remarkably increased in the serum of rats exposed to IR from 119.6 ± 20.6 to 394.4 ± 126.4 compared to the control group (*P* < 0.001). However, a significant decrease was observed in the level of IL-6 in IR + hepatic IPOC (124.4 ± 29.07) and IR + TOFA + hepatic IPOC (182.6 ± 74.97) groups compared to the IR group (394.4 ± 126.4) (*P* < 0.01 and *P* < 0.05, respectively). The increased level of IL-6, AST, and ALT in the hepatic IR rat model shows that the results of the present study are in accord with other studies.^25,26^ We found that the IPOC procedure was able to reduce the release of AST and ALT enzymes in rats exposed to IR. However, tofacitinib as an inhibitor of JAK-STAT was able to inhibit the improvement of liver function induced by IPOC in the liver exposed to IR. While the results of the present study showed that IL-6 was markedly increased in the rats exposed to the IR model, it is evident that the activity of IL-6 is highly correlated with hepatic IRI via IL-6R.^27^ Different studies have reported that IPOC can reduce the level of IL-6 in various organs.^14,18,28,29^ It is shown that the IPOC can enhance the B cell infiltration but reduce the CD8 T cells infiltration into the ischemic organ, thereby, reducing the IL-6 upregulation in the blood of the organism.^28^ The function of IL-6 is crucial for correct homeostasis of hepatic tissue, liver regeneration, infection defense system, and regulation of metabolic functions in the liver.^30^ IL-6 binds to a specific IL-6Rα on the surface of target cells and induces conformational changes in the cytoplasmic domain of gp130 to activate intracellular signaling pathways including PI3K/Akt, ERK1/2, and JAK-STAT.^31^ Consistent with these studies, we found that the IPOC can reduce the serum levels of IL-6 in the animals that underwent the hepatic IR and intriguingly restore it to the control baseline after 24 h of reperfusion.

###  Changes in the expression of the Bax/Bcl-2 ratio in the hepatic tissue

**Figure 2:**
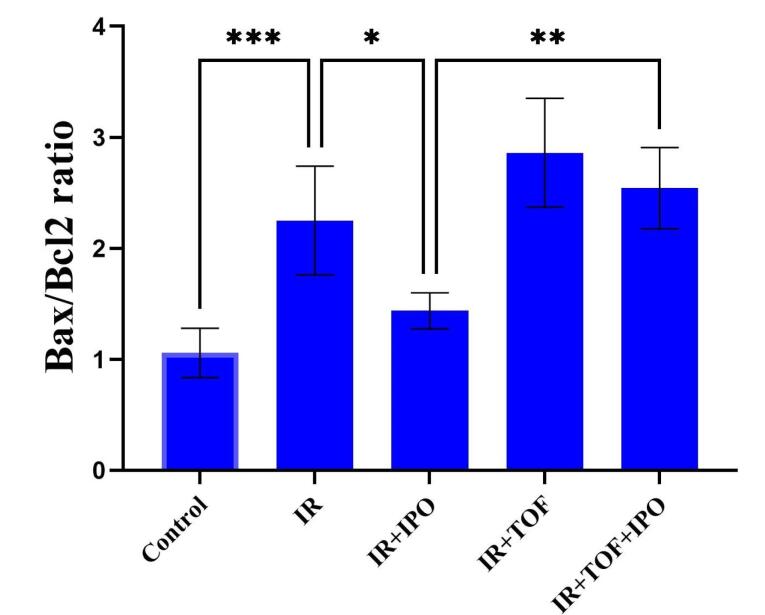
The expression of the Bax/Bcl-2 ratio in the hepatic tissues of different experimental groups that are shown by both **(A) **images and **(B)** Quantification of western blot analysis. Bax/Bcl-2 ratio expressed as the mean ± SD that obtained from at least 5 experiments in each group. * *P* < 0.05, ** *P* < 0.01, *** *P* < 0.001. IR, ischemia/reperfusion; IPOC, Ischemic post-conditioning; TOFA, Tofacitinib

**Figure 3:**
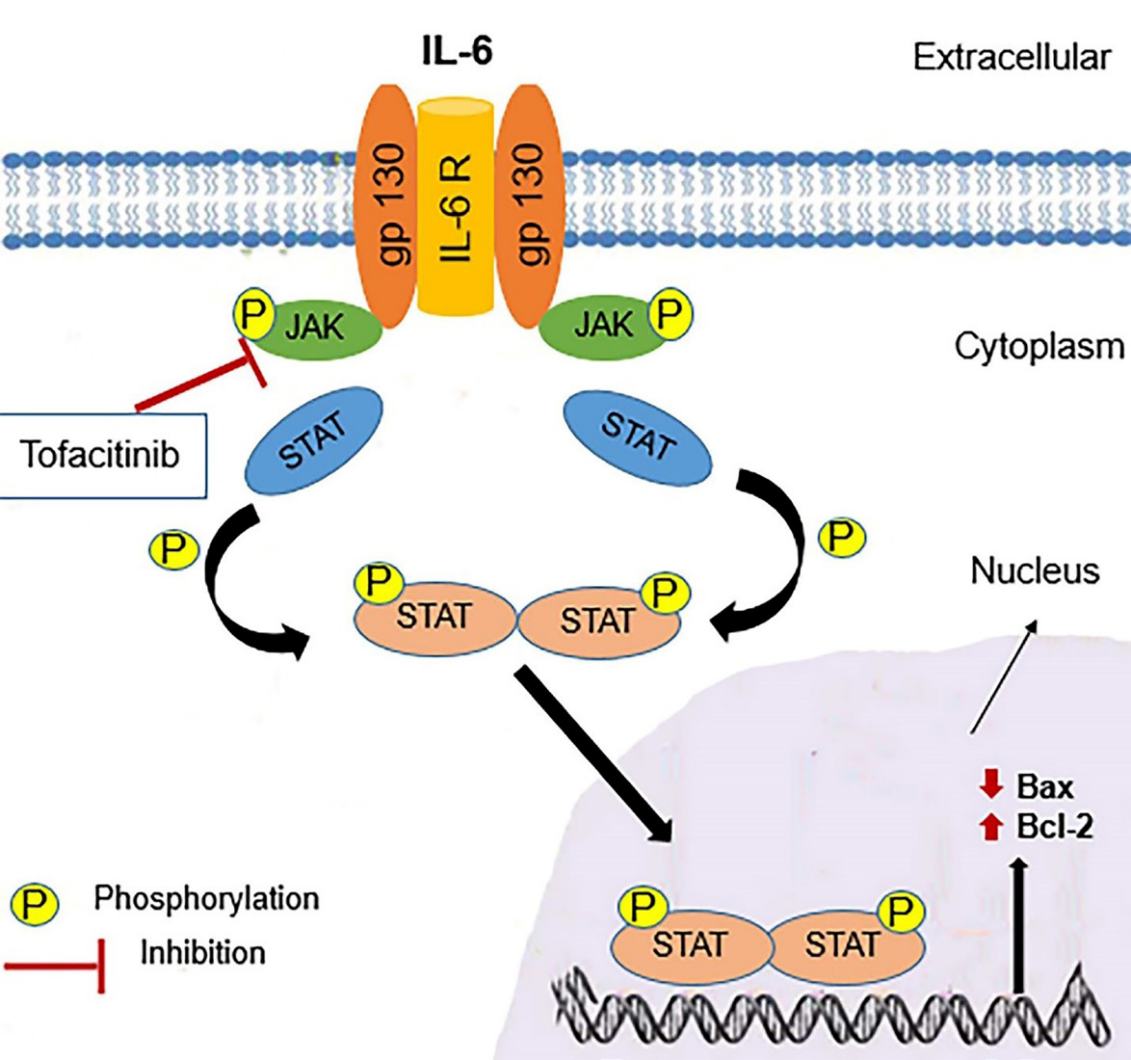
A schematic presentation of the IL-6–JAK-STAT signaling pathway. gp130 is a common receptor chain for IL-6 and the IL-6R which express in Hepatocytes. IL-6 binding to IL-6R followed by the signal is transduced to activate the JAK. JAK phosphorylates the cytoplasmic domain of the receptor to phosphorylation and dimerization of STAT, which translocates to the nucleus and induces the expression of prosurvival factors

 In this study, it is found that treatment of the hepatic IR injury by IPOC enhanced resistance to cell apoptosis as demonstrated by reduced expression of the Bax/Bcl-2 ratio. The representative western blot images and the analysis of data obtained from different groups are shown in Figure 2. As these pictures show, there was a significant increment in the expression of the Bax/Bcl-2 ratio in the group exposed to the IR from 1.05 ± 0.22 to 2.25 ± 0.48 compared to the control group (*P* < 0.001). While there was no significant difference between the IR + hepatic IPOC and control groups, a significant decrease was observed in the Bax/Bcl-2 ratio in the group exposed to IR + hepatic IPOC (1.43 ± 0.16) compared to the IR group (2.25 ± 0.48, *P* < 0.05). The expression of the Bax/Bcl-2 ratio in the IR group exposed to both TOFA and hepatic IPOC was also significantly higher than that in the IR + hepatic IPOC group (2.54 ± 0.36 vs 1.43 ± 0.16, *P* < 0.01). It seems that the injury induced by IR in the liver can be alleviated by the inhibition of apoptosis and related cell death pathways.^32^ Niu et al showed that IPOC in a rat exposed to the hind limb IR reduced the expression of Bax protein and increased the expression of Bcl-2 protein in the hepatic tissue.^33^ In line with this result, Chu et al have demonstrated that IPOC reduced the apoptosis in the hepatic IR conditions via the downregulation of caspase-3 protein and upregulation of cytochrome C in rat hepatocytes.^34^ As our results showed that IPOC was able to restore the increased level of IL-6 in IR-induced rats, it is possible that the IL-6-JAK-STAT pathway could mediate the decrease in the apoptosis level. The IL-6-JAK-STAT3 pathway modulates the expression of multiple genes and proteins involved in the proliferation, differentiation, and survival of different cells,^30^ including Bcl2, Bax, c-myc, Mcl-1, c-FLIP, BAD, MnSOD, Metallothionein (MT1/MT2), and VEGF.^35,36^ Although the IL-6-JAK-STAT3 pathway may play an important role in the IPOC-mediated protection against IR injury in some tissues,^9,37,38^ there is not enough evidence to show clearly the role of this pathway in the context of hepatic IR. Cheng et al also demonstrated that the remote limb IPOC procedure attenuated neuronal apoptosis and inflammation through the JAK-STAT pathway in the rats exposed to focal cerebral ischemia.^37^ The attenuation of the anti-apoptotic effect of IPOC is also shown in a myocardial model of IR by using the AG490 as a JAK2 inhibitor.^38^ Importantly, in the current study, tofacitinib counteracted the anti-apoptotic effects of IPOC in the rat liver exposed to IR injury. Therefore, the results of the present study suggest that the IPOC protection against hepatic IR is possibly mediated through the IL-6-JAK-STAT pathway can emphasize the role and importance of this signaling pathway in health and diseases (Figure 3).

###  Histopathology observations

**Figure 4:**
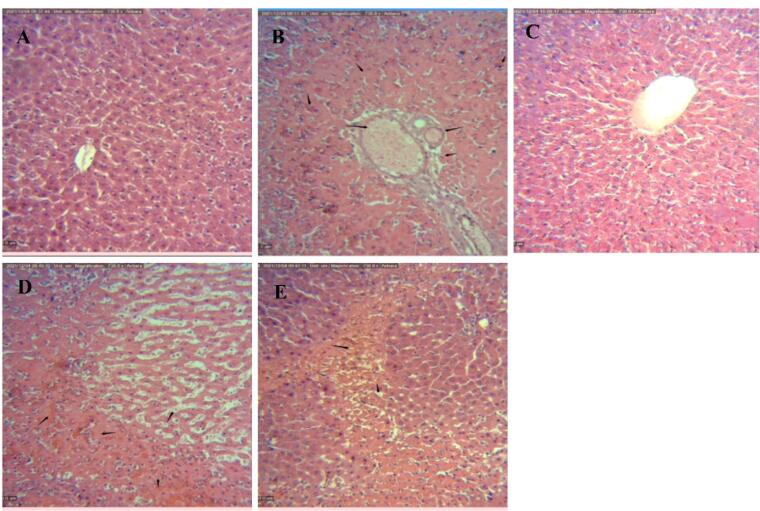
Representative images of hematoxylin and eosin-stained of liver tissues taken from different experimental groups (x100, scale bar 50 µm). **(A)** Control group with no histological changes, **(B)** IR-exposed group illustrating extensive necrosis and fatty infiltration in the hepatocytes, **(C)** IR + IPOC group that show the tiny spot of hepatic necrosis, **(D)** IR + TOFA treated group showing the hemorrhagic necrosis and sinusoidal dilatation in the liver tissues and **(E)** IR + TOFA + IPOC group with extensive necrosis remained in the hepatocytes. IR, Ischemia/reperfusion; IPOC, Ischemic post-conditioning; TOFA, Tofacitinib

 The morphological changes induced by different treatments in the liver sections after 24 h of reperfusion are shown in Figure 4. As these figure shows, while no histological alterations are seen in the sham-operated control group (Figure 4A), a variety of histopathological changes including fat degeneration, hemorrhagic necrosis, and apoptosis were observed in the liver tissues exposed to IR (Figure 4B). However, the numbers and extent of necrotic areas and the occurrence of apoptosis cell death were significantly attenuated in the liver tissues exposed to IPOC (Figure 4C). These histopathological observations can support the biochemical analysis and Western Blot assay that IPOC can restore the normal structure of hepatic tissue and, therefore, ameliorate hepatic dysfunction in IRI. However, the observation obtained from tissue samples taken from the IR group treated with TOFA lonely and/or the TOFA + hepatic IPOC illustrated that some histopathological changes including hemorrhagic necrosis and apoptosis induced by IR remained in these experimental groups (Figures 4D & 4E). These observations are also consistent with other findings of the present study and prove the likely role of the JAK-STAT signaling pathway in the protective effects of hepatic ischemia post-conditioning against the injury induced by ischemia/reperfusion in the rat liver.

## Conclusion

 The results of the present study showed that the IL-6-JAK-STAT signaling pathway may mediate the protective effects of IPOC against the hepatic IR injury as demonstrated by histopathology, IL-6, AST, ALT, and apoptosis assays.

## Acknowledgments

 We appreciate the University of Tehran for financial support. We are also grateful to Dr. Ghorbangol Ashabi and Dr. Aliakbar Golabchifar for friendly providing the necessary materials for this study.

## Competing Interests

 The authors declare there is no conflict of interest.

## Ethical Approval

 The study protocol was reviewed and approved by the ethical committee of the Faculty of Veterinary Medicine, University of Tehran.
